# Mr. Reeve, on a Successful Case of Hydrocephalus

**Published:** 1800-01

**Authors:** R. Reeve

**Affiliations:** Surgeon. West Suffolk Regiment, Chelmsford


### Mr. Reeve, *on a ſucceſsful Caſe of Hydrocephalus.*

### *To the Editors of the Medical and Phyſical Journal.*

Gentlemen,

If the following caſe of Hydrocephalus Internus, which occurred in my own child, ſhould be deemed of ſufficient importance to deſerve a place in your very valuable Journal, the inſertion of it in your earlieſt Number, will much oblige,

Your moſt obedient ſervant,

R. REEVE, Surgeon.

Weſt Suffolk Regiment,

*Chelmsford, Oct.* 29.

The

The fubject of this hiftory, at the age of eight months, in the beginning of December, 1798, could ftand alone, and had every appearance of a healthy, forward child. His temper was unufually placid, and his fpirits invariably good. Towards the end of the month he became extremely coftive, and though medicine for a time relieved him, he was frequently and violently feized with pain in the abdomen, which was generally mitigated by a clyfter. He had at times a great heat, and apparent uneafinefs in the pofterior part of his head, and feemed unable to fupport it; was extremely reftlefs at night, and watchful to an extraordinary degree, all which was fuppofed to arife principally from teeth. From this time he ceafed to grow, except the head, which, towards the end of January, 1799, was perceptibly increafed in fize, and his coftivenefs was become fo obftinate as fcarcely to yield to the moft active purgatives. It was this fingular ftate of the alimentary canal, which had exifted upwards of fix weeks, that firft led me to fufpect fome material derangement in the ftate of the brain. On the 12th of February, he was convulfed in the night, and there was fuch an acceffion of fever that it was thought advifeable to give fmall dofes of Antim. Tartaris. till it fhould have fufficiently cleanfed the primæ viæ; but it produced little or no effect. The following day he took caftor oil, which was repeated a fecond time before any motion was procured; the abdomen was very hard, and of an extraordinary fize; his ftools were of a clay colour, and of fuch an adhefive nature that they could not eafily be feparated from his napkins; his urine was frequently high coloured, fecreted in large quantities, and gave a yellow tinge to his linen. On the 16th, he was put into a warm bath, afterwards wrapped in flannel, and put to bed; a dofe of James's Powder was given him, which occafioned feveral motions of the above defcription. He cried inceffantly towards evening, fhrieked in the moft diftreffing manner, and appeared delirious. His fever now ran very high, pulfe frequent, 130 to 140 in a minute; inceffant thirft; and he had fuch a voracious appetite that he would take with indifference either medicine or food. The next day the warm bath was repeated, and fome neutral falts and abforbent medicines were given, but apparently with no advantage, the fever ftill continuing with unabated fury till the 19th, when a mitigation of his fufferings took place, and for a few hours he appeared perfectly eafy; but at four o'clock the following morning the fever returned, pulfe 130, in which ftate he continued for feveral days, and during that time never clofed his eyes. In the evening of the 21ft he was evidently delirious, his eyes had a moft dazzling brightnefs, and were continually rolling; his cheeks redder than fcarlet. On the the 22d the fever abated a little, but no fleep. On the 23d a drowfinefs came on, which continued uninterruptedly till the 28th; he moaned inceffantly, toffed his head from fide to fide frequently, put his hand up to it, ate voracioufly, but took no notice of any thing. The nature of the complaint was now decided; the increafed fize of the head was very apparent, and the veins running up the left parietal bone extremely varicofe. On the 29th his drowfinefs abated, and he appeared lefs oppreffed. March the 2d, a blifter was applied to the anterior fontanelle, and it was determined to give a grain of calomel twice or three times a day, as the ftomach and bowels were found to bear it; but it was foon obferved to occafion too much pain and irritation to be continued; it was therefore given up for the Ung$^{tm.}$ Hydrarg. fort. of which half a drachm was rubbed in every night. During this time, till the 16th, no material change took place; but his oppreffion was now increafed, and the fever greatly aggravated; he looked death-like pale, moaned much, toffed his head inceffantly from fide to fide, put up his hands to it, coughed violently, vomited a little, and had flight convulfions in the eyelids and mufcles of the mouth. All hope at this time of his recovery was loft; he cried a great deal, had much pain in his bowels, which were diftended by flatus to an alarming degree, and the only relief that could be obtained was by clyfters. He continued in this deplorable ftate till the 26th, with fo little variation that it would be tedious to give the occurrences of each day. The mercurial friction during this period was omitted for a few nights, owing to the exceffive irritation he was in, and neutral falts, carminative and abforbent medicines were given, in order to palliate the moft diftreffing and prevalent fymptoms. The blifter was ftill kept open, which difcharged copioufly at the fontanelle. On the 26th he began to revive again, and to appear eafy and take notice. At this time a profufe perfpiration came on, particularly about the head, which was encouraged by enveloping it in warm flannel. The mercurial frictions were again had recourfe to, and the quantity increafed to two fcruples every night, for the admiffion of which into the fyftem, the moft fcrupulous exactnefs and attention was obferved; for feveral days he continued nearly in the fame ftate. On the 2d of April, his bowels were in excruciating pain, and much diftended with flatulency, though every thing that could be fuggefted, had been done to counteract that tendency. His diet confifted of the moft nutritious broths, with little or no farinaceous matter; his ftrength was fupported (when a ceffation of feverifh fymptoms juftified the exhibition) by a cold infufion of bark and Madeira: he fcreamed for hours inceffantly, and very very frequently alarmed thoſe around him, who expected death every hour as a welcome viſitor; but on the 3d, the alarming ſymptoms were conſiderably abated, and he went on from this time getting progreſſively better, till the 1ſt of May. Great hopes began now to be entertained of his recovery; but on that day he ſhewed great uneaſineſs—the fever returned—his nights were reſtleſs, and though opiates were given, little or no ſleep could be procured. On the 5th, he was taken ſick in the night, had violent pain in his bowels, and was very feveriſh all day. On the 8th, the fever again abated, and he remained without any material change for many days. It was now judged adviſable to diſcontinue the mercury, which was accordingly done, and the bliſter healed on the head, but a ſmall one was opened behind the ear. I cannot date the commencement of his recovery, till the period of dentition, which took place on the 4th of June, when an inciſor of the upper jaw made its appearance. From this time to the 11th, he continued eaſy and cheerful; but now his fever returned—he had reſtleſs nights—cried ſuddenly and violently: theſe ſymptoms, however, abated gradually, and on the 26th, he was taken into the air, enjoyed it much, and ſeemed to mend perceptibly. Colliquative perſpirations continued for ſome time, but at length gradually abated, and he began bathing early in September. His head is reſtored to its natural ſize, and there is no veſtige of diſeaſe remaining in that part, except a ſmall elaſtic projection at the anterior fontanelle, which is more open than it ought to be with a child at his age. I ought to have mentioned, that previous to his illneſs, a ſlight curvature of the ſpine was obſerved, which has increaſed conſiderably with his weakneſs, and renders him at this time, unable to ſit up; but as I conceive this to be a conſequence of an affection of the brain, it will be entirely got the better of, as he acquires ſtrength. The lower extremities were alſo much affected in this diſeaſe. He uſually lies on his back upon the carpet, and is now able to turn himſelf from ſide to ſide with great activity, and is uniformly cheerful and comfortable. His bowels are quite reſtored, and he has left off all medicine. The mercurial friction was continued 35 nights, during which time, two ounces, three drachms, and one ſcruple of the mercurial ointment were rubbed in. It will ſcarcely be credited, but I appeal for the truth of the aſſertion to the teſtimony of Mr. Slater, a ſurgeon of eminence at Margate; a gentleman as much diſtinguiſhed for his humanity, as for his abilities in his profeſſion, under whoſe particular care this very ſingular caſe occurred. And here let me pay that tribute of gratitude to which he is entitled, for his unremitting zeal and conſtant attendance, for upwards upwards of ſix months in the above very diſtreſſing diſeaſe; to whoſe exertions, candor obliges me to acknowledge, I attribute entirely the happy reſult of the caſe. The bliſter was kept open eleven weeks. I have not been particular in mentioning the different medicines preſcribed for the various ſymptoms which occurred from the moment of his firſt attack; as I conceive they had no tendency, till the mercurial plan was adopted, to occaſion re-abſorption of the water in the head. His mouth was never much affected by the mercury, though ſometimes he appeared to have a difficulty in ſwallowing. In this caſe, it was remarkable, that not the ſmalleſt dilatation of the pupils of the eyes was obſerved, through the whole progreſs of the diſeaſe, though he often betrayed much ſenſibility and uneaſineſs, on being ſuddenly expoſed to the light.

